# The Internalized Stigma of Mental Illness (ISMI) scale: validation of the Japanese version

**DOI:** 10.1186/s12888-016-0825-6

**Published:** 2016-04-29

**Authors:** Yosuke Tanabe, Kunihiko Hayashi, Yuki Ideno

**Affiliations:** Department of Laboratory Science and Environmental Health Sciences, Graduate School of Health Sciences, Gunma University, Maebashi, Gunma 371-8514 Japan; Department of Nursing, Takasaki University of Health and Welfare, Takasaki, Gunma 370-0033 Japan; Center for Medical Education, Gunma University, Maebashi, Gunma 371-8511 Japan

**Keywords:** Internalized stigma, Psychometrics, Mental health, Mental disorders, Validation study

## Abstract

**Background:**

The present study investigated the reliability and validity of a Japanese version of the Internalized Stigma of Mental Illness (ISMI) scale, designed to assess internalized stigma experienced by people with mental illness.

**Methods:**

A survey was conducted with 173 outpatients with mental illness who attended psychiatric clinics on a regular basis. A retest was conducted with 51 participants to evaluate the scale’s psychometric properties.

**Results:**

The alpha coefficient for the overall internal consistency was 0.91, and the coefficients of the individual ISMI subscales ranged from 0.57 to 0.81. The test–retest reliability was *r* = 0.85 (*n* = 51, *P* < 0.01). In terms of criterion-related validity, the Japanese version of the ISMI scale was significantly correlated with the Beck Depression Inventory (*r* = 0.61, *P* < 0.01), the Rosenberg Self-Esteem Scale (*r* = −0.53, *P* < 0.01), and the Empowerment Scale (*r* = −0.52, *P* < 0.01). In addition, factor analyses of the ISMI items demonstrated a four-factor solution for the alienation, stereotype endorsement, discrimination experience, and social withdrawal subscales, with the stigma resistance items excluded.

**Conclusions:**

The Japanese version of the ISMI scale demonstrated similar reliability and validity to the original English version. Therefore, the Japanese version of the ISMI scale may be an effective and valid tool to measure internalized stigma among Japanese people who have a mental illness.

**Electronic supplementary material:**

The online version of this article (doi:10.1186/s12888-016-0825-6) contains supplementary material, which is available to authorized users.

## Background

Corrigan proposed a framework that categorized stigma as either public stigma or internalized stigma [1]. “Public stigma” describes the ways in which the general public stigmatizes people with a mental illness, and “internalized stigma” or “self-stigma” represents the internalization of this public stigma [2]. For people with mental illness, internalized or self-stigma is characterized by a subjective perception of devaluation, marginalization, secrecy, shame, and withdrawal [3, 4]. Internalized stigma has a variety of adverse effects, including eroding an individual’s social standing and social networks, and contributing to diminished self-esteem and slower recovery [3, 5]. Internalized stigma is a risk factor for a poorer mental health prognosis [6].

In Japan, prejudice and discrimination against people with mental illness persists. People with mental illness are often isolated under the pretext of hospitalization. The 2011 Patient Survey conducted by the Japanese Ministry of Health, Labour and Welfare found that about 320,000 people with mental illness were hospitalized. This represents 27 hospitalized psychiatric patients per 10,000 people in Japan: the largest number worldwide. Hospitalized people have fewer opportunities for close contact with others, which contributes to a vicious cycle that further delays deinstitutionalization. Moreover, people with mental illness are less likely to find employment than those with physical or intellectual disabilities. Private companies in Japan with more than 50 employees have a 2.0 % employment quota for people with disabilities; these companies employ around 431,225 people with disabilities. Of those employees, 313,314 have physical disabilities, 90,203 have intellectual disabilities, and 27,708 have mental illness (2014 Survey on the Employment Situation of Persons with Disabilities, Japanese Ministry of Health, Labour and Welfare). The self-devaluation, shame, secrecy, and social withdrawal associated with internalized stigma increase the difficulty of overcoming existing barriers to employment [3].

Systematic measurement of internalized stigma provides clinicians and clinical researchers with a confirmable and viable target for general psychotherapeutic interventions. A commonly used measure of internalized stigma is the Internalized Stigma of Mental Illness Scale (ISMI) [3, 7]. The ISMI scale was developed to measure the internalized stigma of people with a mental illness. The scale comprises questions covering the present time and is focused on the respondent’s identity and experience as someone with a mental illness, rather than focusing on hypothetical situations. Worldwide, the ISMI scale has been used as a measure of internalized stigma for people with schizophrenia, depression, leprosy, and AIDS [6, 8–12]. Although the ISMI scale is commonly used to measure internalized stigma, it has not yet been translated into Japanese. Therefore, the present study aimed to test its reliability and validity in a Japanese population.

## Methods

### Participants and procedures

Survey participants were 206 Japanese people who attended eight social welfare facilities in Gunma Prefecture and 13 facilities in Niigata Prefecture in Japan between January and July 2012. The study population comprised individuals with a psychiatric condition that impacted on their ability to function optimally and who were using outpatient mental health services. People attending these facilities are required to submit a medical certificate from a psychiatrist when they register. The presence of mental illness was determined from these medical certificates. We distributed survey material describing the purpose and methods of the study, invitation letters, informed consent forms, and survey questionnaires to participants at the identified facilities. In total, 194 people consented to participate and 12 people refused. Inclusion criteria were use of the selected facilities and ability to understand the Japanese questionnaires. Exclusion criteria were intellectual disability or organic brain disease (*n* = 7) and inability to complete the ISMI questions (*n* = 14). This left 173 participants eligible for study participation. All 173 participants provided informed consent to participate and were surveyed. The study protocol was approved by the Institutional Review Board at Kiryu University.

The participants included 111 males (64.2 %) and 62 females (35.8 %); 62 participants were from eight facilities in Gunma Prefecture (35.8 %) and 111 were from 13 facilities in Niigata Prefecture (64.2 %). These facilities were selected from the teaching facilities for nursing students in Chu-etsu in Niigata Prefecture, and Isesaki City and Kiryu City in Gunma Prefecture. Participants’ self-reported diagnoses were schizophrenia (81.5 %), depression (8.1 %), bipolar disorder (5.8 %), and other psychotic disorders (4.6 %) (Table 1).

**Table 1:** Sample characteristics (*n* = 173)

Characteristic		Mean (S.D.) or % (n)
Age		49.7 years (12.0)
Period of mental illness		24.3 years (12.1)
Gender	Male	64.2 % (111)
	Female	35.8 % (62)
Residence	Gunma Prefecture	35.8 % (62)
	Niigata Prefecture	64.2 % (111)
Education	Junior high school	36.4 % (63)
	High school	41.6 % (72)
	Junior college or vocational school	12.1 % (21)
	University degree or higher	9.8 % (17)
Marital status	Single	75.1 % (130)
	Married	6.4 % (11)
	Divorced or widowed	18.5 % (32)
Cohabitation	Cohabitation	38.2 % (66)
	Living alone	61.8 % (107)
Seif-reported diagnosis	Schizophrenia	81.5 % (141)
	Bipolar disorder	5.8 % (10)
	Depression	8.1 % (14)
	Personality disorder	1.2 % (2)
	Adjustment disorder	0.6 % (1)
	Other	2.9 % (5)
Period of facility use	Less than 6 months	4.0 % (7)
	6 months to less than 1 year	13.9 % (24)
	1 year or more	82.1 % (142)

Retesting was conducted with 55 participants 56–118 days (average 83.1 days) after the initial survey. We excluded responses with missing data and responses indicative of intellectual disability or organic brain disease. Responses from 51 participants were analyzed (92.7 % of the retest sample returned a valid response).

Both the initial and the retest questionnaires were administered to participants in groups of 5–10, and collected on completion.

### Measures

#### Demographic questionnaire

Participants were asked to report their age, sex, place of residence, marital status, level of education, diagnosis, and the age of onset of their illness.

#### Internalized Stigma of Mental Illness (ISMI) scale

The ISMI scale comprises 29 items across five subscales [3]: alienation (6 items), stereotype endorsement (7 items), discrimination experience (5 items), social withdrawal (6 items), and stigma resistance (5 items). The items for each subscale are shown in Table 2. Each item is rated on a four-point Likert scale from 1 (strongly disagree) to 4 (strongly agree). The five stigma resistance subscale items are reverse-coded, and also serve as a validity check [3]. The stigma resistance score is calculated by subtracting the actual value from five. Therefore, stigma resistance displays the same direction of correlation as the other four subscales. A high total score on the ISMI scale indicates more severe internalized stigmatization. The internal consistency of the original version was 0.90 (*n* = 127) and the test–retest reliability coefficient was r = 0.92 (*n* = 16, *P* < 0.05) after a 6-week interval. The internal consistency and test–retest reliability for the five subscales were: alienation, 0.79, 0.68; stereotype endorsement, 0.72, 0.94; discrimination experience, 0.75, 0.89; social withdrawal, 0.80, 0.89; and stigma resistance, 0.58, 0.80 [3].

**Table 2:** Item-level statistics for the ISMI scale (*n* = 173)

Item	Mean	S.D.
Alienation		
I feel out of place in the world because I have a mental illness.	2.3	0.84
I am embarrassed or ashamed that I have a mental illness.	2.3	0.87
I feel inferior to others who don’t have a mental illness.	2.4	0.88
I am disappointed in myself for having a mental illness.	2.3	0.90
Having a mental illness has spoiled my life.	2.3	0.93
People without mental illness could not possibly understand me.	2.4	0.89
Subscale mean	2.35	0.63
Proportion above the midpoint of 2.5	43.1 %	
Stereotype Endorsement		
Mentally ill people tend to be violent.	2.0	0.80
Mentally ill people shouldn’t get married.	1.9	0.81
People with mental illness cannot live a good, rewarding life.	2.1	0.85
People can tell that I have a mental illness by the way I look.	2.1	0.83
Because I have a mental illness, I need others to make most decisions for me.	2.0	0.75
I can’t contribute anything to society because I have a mental illness.	2.2	0.78
Stereotypes about the mentally ill apply to me.	2.4	0.83
Subscale mean	2.10	0.4
Proportion above the midpoint of 2.5	29.7 %	
Discrimination Experience		
People discriminate against me because I have a mental illness.	2.2	0.82
People often patronize me, or treat me like a child, just because I have a mental illness.	2.2	0.79
People ignore me or take me less seriously just because I have a mental illness.	2.3	0.82
Nobody would be interested in getting close to me because I have a mental illness.	2.2	0.76
Others think that I can’t achieve much in life because I have a mental illness.	2.4	0.84
Subscale mean	2.25	0.60
Proportion above the midpoint of 2.5	34.7 %	
Social Withdrawal		
I avoid getting close to people who don’t have a mental illness to avoid rejection.	1.9	0.73
I don’t socialize as much as I used to because my mental illness might make me look or behave “weird”.	2.3	0.86
I don’t talk about myself much because I don’t want to burden others with my mental illness.	2.5	0.85
Negative stereotypes about mental illness keep me isolated from the “normal” world.	2.4	0.83
Being around people who don’t have a mental illness makes me feel out of place or inadequate.	2.3	0.78
I stay away from social situations in order to protect my family or friends from embarrassment.	2.2	0.78
Subscale mean	2.27	0.56
Proportion above the midpoint of 2.5	37.8 %	
Stigma Resistance (reverse-coded items)		
People with mental illness make important contributions to society.	2.8	0.80
I feel comfortable being seen in public with an obviously mentally ill person.	3.1	0.71
Living with mental illness has made me a tough survivor.	2.5	0.80
In general, I am able to live my life the way I want to.	2.7	0.82
I can have a good, fulfilling life, despite my mental illness.	2.7	0.83
Subscale mean	2.75	0.48
Proportion above the midpoint of 2.5	63.5 %	
Total scale mean	2.33	0.43
Proportion above the midpoint of 2.5	40.8 %	

The original English version of the ISMI scale was translated into Japanese, and then back-translated into English by two people with a good command of English. This back-translation was sent to the authors of the original English version to obtain their opinion as to its suitability. This back-translation process was repeated five times, with Ritsher and colleagues approving the final translation for use as the Japanese version of the ISMI scale Additional file 1.

#### External validity

Three scales were identified to test criterion validity based on previous research [3].

1. *Beck Depression Inventory (BDI)*

The BDI is a subjective self-evaluation scale comprising 21 items measuring depressive symptoms [13, 14] and is commonly used in Japan. Each item is evaluated on a scale from 0 (none) to 3 (severe), with the total score ranging from 0 to 63. The severity of depressive symptoms is determined from the total score, with higher scores indicating more severe symptoms. Mean scores and standard deviations (SD) were 12.6 (SD = 9.9, *n* = 120), and the internal consistency reliability was 0.93 [14].

2. *Rosenberg Self-Esteem Scale (RSES)*

Self-esteem was measured using the RSES [15, 16]. RSES items are evaluated on a four-point Likert scale from 1 (strongly disagree) to 4 (strongly agree). The total score ranges from 4 to 40, with higher scores reflecting higher levels of self-esteem. The reliability and validity of the Japanese version of the RSES were verified by Uchida et al. [17].

3. *Empowerment Scale (ES)*

Empowerment refers to an individual’s power with respect to their own daily life and to the living environment and society in which they are placed [18–20]. This represents a motivated attitude, and the polar opposite of internalized stigma. The ES, developed by Rogers et al., is a self-administered scale that evaluates the mental aspects of empowerment, and was designed for people with mental illness [18]. The ES consists of 28 questions, evaluated on a four-point Likert scale from 1 (strongly agree) to 4 (strongly disagree). The total score ranges from 28 to 112; a higher score indicates a higher level of empowerment. ES items include “I have a positive attitude about myself,” “I feel powerless most of the time,” and “People have more power if they join together as a group.” The reliability and validity of the Japanese version of the ES were verified by Hata et al. [21].

### Data analysis

The reliability of the Japanese version of the ISMI scale was evaluated by testing the internal consistency of the items using Cronbach’s alpha, and by the scale’s test–retest reliability using Pearson’s correlation coefficients (*r*) between total scores. The validity of the scale was investigated through criterion-related validity and construct validity. Criterion-related validity was verified through the correlation of the ISMI scale with the BDI, the RSES, and the ES. Construct validity was investigated using maximum likelihood factor analysis with a promax rotation of 24 items in four subscales, excluding the stigma resistance subscale. As a result of factor analysis of the ISMI scale and other scales, Ritsher et al. were able to sort 24 items (excluding the five stigma resistance subscale items) onto the expected factor structure. The factor analyses in the present study were performed in the same manner as in Ritsher et al. [3]. The eigenvalues-greater-than-1.0 rule was applied to clarify the number of factors extracted. The differences in total ISMI score by sex, place of residence, education, marital status, cohabitation, and period of facility use were investigated using t-tests and one-way analysis of variance. All analyses were performed using SPSS Version 21.0 (IBM SPSS Statistics for Windows, Version 21.0. Armonk, NY: IBM Corp.).

## Results

### Total ISMI scores

Mean scores and SDs were obtained for the five subscales: alienation, 2.35 (SD = 0.63); stereotype endorsement, 2.10 (SD = 0.48); discrimination experience, 2.25 (SD = 0.60); social withdrawal, 2.27 (SD = 0.56); and stigma resistance, 2.75 (SD = 0.48). The means of the ISMI subscale scores and the proportion of participants above the midpoint (2.5) are shown in Table 2. There were no statistically significant differences in ISMI mean total scores for sex, residence area, education, marital status, cohabitation, and period of facility use. There was no statistically significant difference in ISMI mean total score for self-reported diagnosis (Table 3).

**Table 3:** Total ISMI scores by demographics (*n* = 173)

		Mean total score	Test
Gender	Male	2.30	*t* = −1.09
	Female	2.38	(*p* = 0.28)
Residence	Gunma Prefecture	2.28	*t* = −1.00
	Niigata Prefecture	2.35	(*p* = 0.32)
Education	Junior high school	2.33	*F* = 0.57
	High school	2.29	(*p* = 0.97)
	Junior college or vocational school	2.41	
	University degree or higher	2.39	
Marital status	Single	2.37	*F* = 2.06
	Married	2.22	(*p* = 0.86)
	Divorced or widowed	2.21	
Cohabitation	Cohabitation	2.35	*t* = 0.42
	Living alone	2.32	(*p* = 0.67)
Period of facility use	Less than 6 months	2.42	*F* = 0.73
	6 months to less than 1 year	2.41	(*p* = 0.79)
	1 year or more	2.31	
Self-reported diagnosis	Schizophrenia	2.33	
	Bipolar disorder	2.22	*F* = 0.22
	Depression	2.34	(*p* = 0.04)
	Other	2.38	

### Reliability

#### Internal consistency

The alpha coefficient for the internal consistency of the overall scale was 0.88. Table 4 shows the Cronbach’s alphas for each subscale in the Japanese version of the ISMI scale, alongside those obtained for the original English version. The alphas for each subscale in the Japanese version ranged from 0.57 to 0.81; the lowest coefficient was 0.57 for the stigma resistance subscale.

**Table 4:** Cronbach’s alpha coefficients in relation to the internal consistency of the ISMI subscales (*n* = 173)

Subscale	Japanese version Cronbach’s alpha	Original English version Cronbach’s alpha
Alienation	0.81	0.79
Stereotype endorsement	0.70	0.72
Discrimination experience	0.80	0.75
Social withdrawal	0.79	0.80
Stigma resistance	0.57	0.58

#### Test–retest reliability

Significant correlations were found for all subscale scores: total score, *r* = 0.85 (*P* < 0.01); alienation, *r* = 0.83 (*P* < 0.01); stereotype endorsement, *r* = 0.79 (*P* < 0.01); discrimination experience, *r* = 0.79 (*P* < 0.01); social withdrawal, *r* = 0.73 (*P* < 0.01); and stigma resistance, *r* = 0.71 (*P* < 0.01).

### Validity

#### Criterion-related validity

The ISMI subscales were positively correlated with the BDI and negatively correlated with the RSES and the ES. The ISMI total scores were correlated with the BDI (*r* = 0.61, *P* < 0.01), the RSES (*r* = —0.53, *P* < 0.01), and the ES (*r* = —0.52, *P* < 0.01). Table 5 shows the correlations between the ISMI scores and the scores for the other three scales. All correlations were significant (*P* < 0.01).

**Table 5:** Correlation between ISMI and Beck depression inventory, Rosenberg self-esteem scale, and empowerment scale scores (*n* = 173)

ISMI	BDI	RSES	ES
Alienation	0.59**	−0.49**	−0.43**
Stereotype endorsement	0.50**	−0.42**	−0.45**
Discrimination experience	0.56**	−0.47**	−0.47**
Social withdrawal	0.45**	−0.37**	−0.37**
Stigma resistance	0.23**	−0.31**	−0.31**
Total score	0.61**	−0.53**	−0.52**

**P < 0.01

#### Construct validity

Table 6 shows the factor analysis for the 24 items in four subscales (excluding the stigma resistance subscale). Four factors were obtained. The first factor included almost all of the social withdrawal items. The second factor included the alienation and stereotype endorsement items, with alienation showing a higher loading. The third factor included almost all discrimination experience items. The fourth factor included the stereotype endorsement items.

**Table 6:** Factor analysis of items from four main ISMI subscales (excluding the Stigma Resistance subscale) (*n* = 173)

Item	Social withdrawal	Alienation	Discrimination experience	Stereotype endorsement
I am embarrassed or ashamed that I have a mental illness	**0.79**	−0.03	−0.04	−0.08
I feel inferior to others who don’t have a mental illness	**0.63**	0.08	0.04	−0.06
I don’t talk about myself much because I don’t want to burden others with my mental illness	**0.60**	0.01	−0.14	0.24
I feel out of place in the world because I have a mental illness	**0.59**	−0.11	0.24	−0.06
Being around people who don’t have a mental illness makes me feel out of place or inadequate	**0.55**	0.14	0.08	0.07
Mentally ill people tend to be violent	**0.49**	−0.06	0.13	0.07
I don’t socialize as much as I used to because my mental illness might make me look or behave “weird”	**0.46**	0.22	0.05	0.07
I avoid getting close to people who don’t have a mental illness to avoid rejection	**0.45**	−0.08	0.10	0.09
I stay away from social situations in order to protect my family or friends from embarrassment	0.32	0.14	−0.04	0.14
I am disappointed in myself for having a mental illness	0.04	**0.75**	0.07	−0.01
People with mental illness cannot live a good, rewarding life	0.21	**0.68**	−0.06	−0.21
I can’t contribute anything to society because I have a mental illness	−0.24	**0.67**	0.19	0.15
Having a mental illness has spoiled my life	−0.02	**0.65**	0.31	−0.17
Others think that I can’t achieve much in life because I have a mental illness	0.19	**0.40**	0.15	0.12
People often patronize me, or treat me like a child, just because I have a mental illness	−0.08	0.03	**0.67**	0.18
People discriminate against me because I have a mental illness	0.34	−0.12	**0.61**	−0.10
People ignore me or take me less seriously just because I have a mental illness	-0.10	0.25	**0.54**	0.21
People without mental illness could not possibly understand me	0.06	0.24	**0.54**	−0.04
Negative stereotypes about mental illness keep me isolated from the “normal” world	0.41	0.07	**0.43**	−0.16
Mentally ill people shouldn’t get married	0.31	0.31	−0.39	0.15
Nobody would be interested in getting close to me because I have a mental illness	0.05	0.24	0.30	0.18
People can tell that I have a mental illness by the way I look	0.06	−0.28	0.27	**0.67**
Because I have a mental illness, I need others to make most decisions for me	0.01	0.11	−0.08	**0.55**
Stereotypes about the mentally ill apply to me	0.24	0.13	0.04	0.36
Eigenvalue	8.739	1.669	1.458	1.285
Cumulative contribution ratio	0.364	0.434	0.494	0.548

Figures in bold show factor loadings > 0.40

## Discussion

The results of the present study demonstrate that the Japanese version of the ISMI scale has good reliability and validity. The reliability was evaluated by testing the internal consistency of the items, and by the test–retest reliability of the scale. The validity of the scale was investigated with criterion-related validity and construct validity. We obtained similar results to the original scale for internal consistency, test–retest reliability, criterion-related validity, and construct validity.

Analysis of the internal consistency of the Japanese version of the ISMI scale supported the reliability of the scale. The subscale with the lowest internal consistency was stigma resistance, which was consistent with the original version of the ISMI scale. The internal consistency was similar when we compared other subscales. In addition, the overall internal consistency of the Japanese version of the ISMI scale was similar to that of the original English version.

The test–retest reliability coefficient for the present study was similar to the value for the original English version [3]. The correlations were also close and may not be significantly different from each other. Therefore, the Japanese version of the ISMI scale demonstrated a similar level of reliability to the original version. The average test–retest interval was 83.1 days (range 56–118 days); we could have conducted a test–retest study using shorter and more consistent intervals. However, in other test–retest studies with stigma scales, the study intervals were 2 weeks [22], 5 weeks [23], 6 weeks [3], and 4 months [24]; our test–retest interval was in the range of those studies. In our test–retest study population, the ISMI scores were relatively stable over time.

Our results for criterion-related validity were consistent with those obtained for the original English version of the ISMI scale; the BDI showed a positive association with the ISMI scale, and the RSES and the ES showed negative associations. In general, tendencies toward stigma and depressive symptoms are negatively-themed constructs, while self-esteem and empowerment are positive constructs. The correlations obtained in the present study represent strong evidence for the criterion-related validity of the Japanese version of the ISMI scale.

In our examination of construct validity, we also obtained the same result as for the original English version. Four factors were obtained from the factor analysis: the first factor was social withdrawal, the second was alienation, the third was discrimination experience, and the fourth factor was stereotype endorsement. Although the first factor included both social withdrawal and alienation items, there were more social withdrawal items. This was consistent with the results of the factor analysis of the original version of the ISMI scale. Therefore, the Japanese version of the ISMI scale appears to have sufficient construct validity. In general, Japanese people do not like direct expression. Therefore, in our translation, we used expressions to represent the scale items that were as sensitive as possible. As a result, the translation was not an exact match. By repeating the back-translation a number of times, we were able to develop a Japanese version of the ISMI scale that was consistent with the versions used in other countries. That is, the final Japanese expressions used reflected the original questions well.

To examine differences across countries in ISMI subscale scores of people with schizophrenia, we compared our findings with those from eight other studies [8, 25–31]. There were no large differences in mean scores on the stigma subscales across the countries reviewed (Table 7). The means for Japan were low, and the scoring profile (except for stigma resistance) resembled findings from China, also located in East Asia. Ersoy et al. found that the total score differed significantly by sex and income when testing the Turkish version of the ISMI scale: men had higher scores than women, and individuals with low or moderate income levels had higher scores than those with higher incomes [10]. The effects of sex, education, and other demographic variables on the ISMI scale scores were not reported in the original English version. In the present study, there were no significant differences in the association between the total ISMI scale score and demographic variables. This indicates that the Japanese version of the ISMI scale can be used with diverse populations with different demographics.

**Table 7:** Comparison of mean scores of stigma domains between patients with schizophrenia in different countries

	Country/region	n	Alienation	Stereotype endorsement	Discrimination experience	Social withdrawal	Stigma resistance
The present study	Japan	141^a^	2.35 ± 0.88	2.12 ± 0.83	2.25 ± 0.80	2.28 ± 0.83	2.75 ± 0.83
Lv et al. [25]	China	95	2.29 ± 0.49	2.07 ± 0.44	2.15 ± 0.45	2.19 ± 0.46	2.17 ± 0.38
Brohan et al. [26]	14 European	1220^b^	2.53 ± 0.70	2.19 ± 0.53	2.43 ± 0.61	2.48 ± 0.66	2.47 ± 0.51
Sibitz et al. [27]	Austria	157	2.28 ± 0.86	1.87 ± 0.63	2.16 ± 0.79	2.13 ± 0.74	2.73 ± 0.76
Lysaker et al. [28]	US	133	2.45 ± 0.68	2.00 ± 0.51	2.48 ± 0.65	2.37 ± 0.65	2.25 ± 0.51
Lysaker et al. [29]	US	75	2.31 ± 0.65	1.99 ± 0.54	2.42 ± 0.69	2.30 ± 0.66	2.17 ± 0.52
Lysaker et al. [30]^c^	US	36	1.39 ± 0.68	1.14 ± 0.61	1.42 ± 0.70	1.31 ± 0.67	1.24 ± 0.50
Assefa et al. [31]	Ethiopia	212	2.3 ± 0.6	2.5 ± 0.6	2.5 ± 0.7	2.4 ± 0.9	-
Botha et al. [8]	South African	100	2.50	2.16	2.90	2.63	-

^a^ Patients with schizophrenia

^b^ Including patients with other mental disease

^c^ The authors used a 0-3 scale for the ISMI

There are some limitations in our study, including a potential response bias in the test–retest because of the test and retest timing. In addition, the participants in the present study were outpatients who used social welfare facilities, and 81.5 % were patients with schizophrenia. The nationwide Japanese Patients Survey conducted by the Japanese Ministry of Health, Labour and Welfare found that 18.7 % of outpatients and 58.5 % of inpatients with a mental illness had schizophrenia. Therefore, our study population may not be representative of the Japanese population with a mental illness. Moreover, we did not measure cultural or ethnographic variables such as religion.

In a pilot study, we found that some participants were not able to complete the questionnaire by themselves. Therefore, in the present study, participants received guidance in the form of a researcher who read questions and answer selections aloud and waited for participants’ responses. Therefore, the reliability and validity of the scale self-administered without this guidance may differ from the present study.

## Conclusions

The results of the present study demonstrated that the Japanese version of the ISMI scale has similar levels of reliability and validity to the original English version. Therefore, the Japanese version of the ISMI scale may be used as a valid tool to reliably measure internalized stigma in Japanese people who have a mental illness. We hope that this scale will be of use to clinicians and researchers in Japan to advance understanding of the internalized stigma experienced by people who have a mental illness.

### Ethics approval and consent to participate

The study was conducted after the approval of the study protocol by Kiryu University Institutional Review Board. Participation was voluntary and information was collected anonymously after obtaining written consent from each respondent by assuring confidentiality throughout the data collection period.

### Consent for publication

Not applicable.

### Availability of data and materials

Data and materials supporting our findings in the manuscript will not be shared. It was not in accordance with participants’ written informed consent.

## Additional files

Additional file 1: The Internalized Stigma of Mental Illness (ISMI) Scale, Japanese version. (DOCX 37 kb)
