# Supplementary material for: The Internalized Stigma of Mental Illness (ISMI) scale: validation of the Japanese version
Source: BMC Psychiatry. 2016 Apr 29;16:116. doi: 10.1186/s12888-016-0825-6 (PMC4850681; doi:10.1186/s12888-016-0825-6)
Supplement: Additional file 1: — The Internalized Stigma of Mental Illness (ISMI) Scale, Japanese version. (DOCX 37 kb) [file 12888_2016_825_MOESM1_ESM.docx]

Additional file 1. The Internalized Stigma of Illness(ISMI) Scale, Japanese version

精神障がい者の内面化したスティグマ尺度

このアンケートにおいて、「精神疾患」という言葉を使用しておりますが、あなたがもっと好ましく感じる言葉があれば、それに置き換えて考えてみてください。

それぞれの質問に対して、（１）全くそう思わない、（２）そう思わない、（３）そう思う、（４）非常にそう思う、のどれかを選んで、文章の右にある数字のうち１つを「○」で囲んでください。

４　非常にそう思う

３　そう思う

２　そう思わない

１　全くそう思わない

| 1. | 私は精神疾患にかかっているため、世の中から除外されているように感じる。 | 1 | 2 | 3 | 4 |
| --- | --- | --- | --- | --- | --- |
| 2. | 精神疾患の人は暴力的になりがちである。 | 1 | 2 | 3 | 4 |
| 3. | 私が精神疾患なので、人々は私を差別する。 | 1 | 2 | 3 | 4 |
| 4. | 私は拒絶されないために、精神疾患ではない人と親しくなるのを避ける。 | 1 | 2 | 3 | 4 |
| 5. | 私は精神疾患であることをとまどったり、または恥じたりしている。 | 1 | 2 | 3 | 4 |
| 6. | 精神疾患の人は結婚すべきではない。 | 1 | 2 | 3 | 4 |
| 7. | 精神疾患をもった人は、社会に重要な貢献を  する。 | 1 | 2 | 3 | 4 |
| 8. | 精神疾患ではない人に、私は劣等感を感じる。 | 1 | 2 | 3 | 4 |
| 9. | 私が精神疾患であるため、「気味悪く」見えるか,または振る舞うかもしれないので、以前ほど社交的ではない。 | 1 | 2 | 3 | 4 |
| 10. | 精神疾患をもった人は、りっぱな、価値のある  人生を送ることはできない。 | 1 | 2 | 3 | 4 |
| 11. | 私が精神疾患であることで、他の人に負担を  かけたくないので、私は自分自身について  あまり話さない。 | 1 | 2 | 3 | 4 |
| 12. | 精神疾患に対する社会一般で通用している  否定的な思い込みが、｢正常｣な世界から私を孤立させている。 | 1 | 2 | 3 | 4 |
| 13. | 精神疾患ではない人といると、場違いとか、  または不適格だと自分自身で感じてしまう。 | 1 | 2 | 3 | 4 |
| 14. | 私は、明らかに精神的に病んでいる誰かと一緒に、おおぜいの前で見られることを心地よく  感じる。 | 1 | 2 | 3 | 4 |
| 15. | 私が精神疾患なので、しばしば人は私に恩着せがましい態度をとるか、あるいは私を子供扱いする。 | 1 | 2 | 3 | 4 |
| 16. | 精神疾患であるため、私は自分自身に失望している。 | 1 | 2 | 3 | 4 |
| 17. | 精神疾患があることで、私の人生はダメにさせられた。 | 1 | 2 | 3 | 4 |
| 18. | 人は私の外見から、私が精神疾患にかかっていると、見分けることができる。 | 1 | 2 | 3 | 4 |
| 19. | 私は精神疾患なので、他の人に、私のための  ほとんどのことを決定してもらう必要がある。 | 1 | 2 | 3 | 4 |
| 20. | 家族や友達をとまどいから守るために、私は  交流の場から遠のいている。 | 1 | 2 | 3 | 4 |
| 21. | 精神疾患ではない人は、どうしても私を理解  できない。 | 1 | 2 | 3 | 4 |
| 22. | 私が精神疾患なので、人は私を無視するか、  私をそれほど真剣には受け止めない。 | 1 | 2 | 3 | 4 |
| 23. | 私は精神疾患なので、社会に何らかの貢献をすることはできない。 | 1 | 2 | 3 | 4 |
| 24. | 精神疾患でありながら、生きていくことは、私をたくましく困難を切り抜けていく人にしている。 | 1 | 2 | 3 | 4 |
| 25. | 私が精神疾患なので、私に近づくことに、誰も興味をもたないだろう。 | 1 | 2 | 3 | 4 |
| 26. | おおざっぱに言うと、私がやりたいように、自分の人生を生きることができる。 | 1 | 2 | 3 | 4 |
| 27. | 精神疾患にもかかわらず、私はすぐれた満ち  たりた人生を送ることができる。 | 1 | 2 | 3 | 4 |
| 28. | 私が精神疾患なので、他の人は私が人生で  何かを達成することはできないと思っている。 | 1 | 2 | 3 | 4 |
| 29. | 精神疾患に対する、社会一般の型にはまった思い込みは、私にもあてはまる。 | 1 | 2 | 3 | 4 |
